# POLE and Mismatch Repair Status, Checkpoint Proteins and Tumor-Infiltrating Lymphocytes in Combination, and Tumor Differentiation: Identify Endometrial Cancers for Immunotherapy

**DOI:** 10.3389/fonc.2021.640018

**Published:** 2021-03-19

**Authors:** Dandan Dong, Huajiang Lei, Duanya Liu, Hansong Bai, Yue Yang, Baijie Tang, Ke Li, Juan Liu, Gang Xu, Xue Xiao

**Affiliations:** ^1^ Department of Pathology, Sichuan Provincial People’s Hospital, University of Electronic Science and Technology of China, Chengdu, China; ^2^ Chinese Academy of Sciences Sichuan Translational Medicine Research Hospital, Chengdu, China; ^3^ Department of Gynecology and Obstetrics, Sichuan Provincial People’s Hospital, University of Electronic Science and Technology of China, Chengdu, China

**Keywords:** endometrial cancer, mismatch repair deficiency, immunotherapy, POLE, PD-1, PD-L1

## Abstract

**Objective:**

Although Polymerase-epsilon (POLE)-mutated and mismatch repair (MMR)-deficient endometrial cancers (ECs) are considered as promising candidates for anti-PD-1/PD-L1 therapy, selecting only these patients may exclude other patients who could potentially respond to this treatment strategy, highlighting the need of additional biomarkers for better patient selection. This study aims to evaluate potential predictive biomarkers for anti-PD-1/PD-L1 therapy in addition to POLE mutation (POLEm) and MMR deficiency (MMRd).

**Methods:**

We performed next generation sequencing for POLE from 202 ECs, and immunohistochemistry for MLH1, MSH2, MSH6, PMS2, CD3, CD8, PD-1 and PD-L1 on full-section slides from these ECs. We assessed the association of POLEm and MMRd with clinicopathologic features, expression of check point proteins, and density of tumor-infiltrating lymphocytes (TILs). Prognostic impact of these immune markers was also evaluated.

**Results:**

POLEm, MMRd and high-grade tumors exhibited elevated level of TILs. Increased expression of PD-1 and PD-L1 was observed in MMRd and high-grade ECs. A subgroup of MMR proficient ECs also harbored increased density of TILs, and positive expression of PD-1 and PD-L1. In addition, negative expression of checkpoint proteins and high density of TILs in combination was associated with good prognosis.

**Conclusions:**

Candidates for PD-1 blockade may extend beyond POLEm and MMRd ECs, additional factors such as tumor grade, and combination of TILs levels and expression of checkpoint proteins may need to be considered for better patient selection.

## Introduction

Endometrial carcinoma (EC) is the most common gynecological malignancy in the developed world, and affects over 300,000 women worldwide annually (1–3). Traditionally, ECs have been classified as either endometrioid (Type I) or non-endometrioid (Type II) types based on clinical and histopathological criteria (4). ECs can also be classified into four distinct molecular subgroups, two of which are associated with high mutational load: ultra-mutated and hyper-mutated (4, 5). Ultra-mutated tumors harbor mutations in the exonuclease domain of the polymerase epsilon (POLE), while hyper-mutated tumors are characterized by microsatellite instability-high (MSI-H) resulting from deficiency of mismatch repair proteins (5). POLE proofreading mutations (POLEm) and mismatch repair deficiency (MMRd) lead to high numbers of DNA replication errors and high mutation frequency. The abnormal peptides generated by these tumors, known as neoantigens, have been shown to elicit host immune response, are potential targets for immunotherapy (6–8).

In recent years, immunotherapy has revolutionized the treatment of patients with various cancer types, such as melanoma, non-small cell lung cancer, and most notably MSI-H/MMRd tumors irrespective of tumor type (9–11). However, response to immunotherapy is complex and durable benefit is limited to a small subset of patients, highlighting the need for predictive biomarkers (10). POLEm, MMRd, and expression of checkpoint proteins appear to be related but their association with one another in EC remain unclear. In addition, besides MMRd tumors, a subset of mismatch repair proficient (MMRp) ECs had objective responses to combination treatment of pembrolizumab and lenvatinib, suggesting additional factors of immunogenicity need to be taken into consideration to predict response of immunotherapy (12, 13). Moreover, the value of checkpoint proteins as prognostic markers in EC is under-investigated. Therefore, we performed targeted next generation sequencing (NGS) and immunohistochemistry (IHC) in a series of ECs, and evaluated the association of POLEm and MMRd with clinicopathologic characteristics, expression of PD-1 and PD-L1, and density of CD3+ and CD8+ tumor-infiltrating lymphocytes (TILs). We also assessed the prognostic impact of POLEm, MMRd, TILs and checkpoint proteins. Our findings suggest that candidates for anti-PD-1/PD-L1 therapy extend beyond POLEm and MMRd ECs, and assessment of additional markers such as tumor differentiation, level of CD3+ and CD8+ TILs, and expression of PD-1 and PD-L1 may be helpful for patient selection. In addition, our study supports using checkpoint proteins (PD-1 or PD-L1) and TILs (CD3+ or CD8+) as combined prognostic markers for EC.

## Materials and Methods

### Tumor Samples and Clinical Data

The retrospective study was approved by the ethics committee of Sichuan Provincial People’s Hospital (Chengdu, China). Formalin-fixed, paraffin-embedded (FFPE) tumor blocks of 228 consecutive ECs diagnosed between January 2013 and November 2016 were retrieved from the department of pathology. Hematoxylin and eosin (H&E) staining was performed on 4-μm fresh sections of all samples. Pathological diagnoses were re-confirmed by two pathologists. Clinicopathologic characteristics and follow-up information were obtained from electronic medical records. Overall survival (OS) was defined as the length of time between the date of surgery and death (any cause). Progression free survival (PFS) was defined as the time between the date of surgery and disease progress/relapse.

### Identification of POLE-Mutated Tumors

DNA of the 228 tumors was extracted from FFPE samples. Twenty six patients were excluded for further experiments because of poor DNA quality. For the remaining 202 tumors, mutations in the exonuclease domain of POLE (exons 9–14) was identified by targeted NGS. A set of primers were designed using Primer 3 to cover exons 9–14 of *POLE* (NM_006231). The amplification reactions were carried out using Applied Biosystems 2720 Thermal Cycler (Life Technologies Corporation, USA). DNA barcodes (8 bp) were added to the PCR products, and all the libraries of each sample were pooled. After cluster generation and hybridization of sequencing primer, the library pools were sequenced using Hiseq2500 sequencing system (Illumina, Inc, San Diego, CA), with an average depth of coverage >1,000×. The functional effect of mutations was assessed by *in silico* prediction tools: Non-synonymous single nucleotide variations (SNVs) deemed pathogenic by at least two of the three algorithms (SIFT, PolyPhen-2 and Mutation Taster), and pathogenic mutations in splice sites predicted by both AdaBoost and Random Forest were included for further analysis (14–17).

### Immunohistochemistry

IHC was performed on whole-slide sections from the 228 FFPE tumor blocks. The serial sections were incubated at 60°C for 1 h following deparaffinization by Xylol and rehydration by a series of descending concentrations of alcohol. After heat induced antigen retrieval, slides were blocked in 3% H_2_O_2_, then stained for MLH1 (clone ES05), MSH2 (clone RED2), MSH6 (clone EP49), PMS2 (clone M0R4G), CD3 (clone SP7), CD8 (clone SP16), PD-1 (clone UMAB199) and PD-L1 (clone SP142) (**supplementary data1**). After incubation with secondary antibody (30 min) and DAB development (Dako REAL™ EnVision™ Detection System), slides were counterstained with hematoxylin and coverslipped.

### Determination of MMR Status

MMR status of each sample was determined by IHC of MMR proteins (MLH1, MSH2, MSH6, and PMS2). Nuclear staining of lymphocytes, stromal cells or normal endometrium was used as positive internal controls. Mismatch repair deficiency (MMRd) was defined as complete loss of nuclear staining of any MMR protein in tumor cells with presence of positive internal controls. Tumors showed expression of all four MMR proteins were defined as mismatch repair proficiency (MMRp).

### Assessment of TILs and Expression of PD-1 and PD-L1

The assessment of TILs and expression of PD-1 and PD-L1 was conducted with blinding to POLE and MMR status. The density of CD3, CD8 or PD-1 was evaluated as the number of CD3+, CD8+ or PD-1+ lymphocytes located within tumor epithelium. For each sample the average count was determined from five randomly selected high-power fields (HPF). Tumors with an average of one or greater PD-1+ TIL per HPF were considered PD-1 positive (18). PD-L1 expression in tumor cells (TC) was scored based on the proportion of tumor area occupied by PD-L1 expression tumor cells (membranous staining) of any intensity. PD-L1 expression in immune cells (IC) was scored based on the proportion of tumor area occupied by PD-L1 staining immune cells of any intensity. Positive TC expression of PD-L1 was defined as TC score ≥1%, while positive IC expression of PD-L1 was defined as IC score ≥5% (19–22). For survival analysis, tumors with PD-L1 TC positive or/and PD-L1 IC positive were defined as PD-L1 positive.

### Statistical Analysis

After targeted NGS and IHC, 202 cases with defined POLE and MMR status were included in statistical analysis. Data were analyzed using GraphPad Prism 7. Fisher’s exact test and Chi-square test were used for cross-tables. Unpaired t test, Mann–Whitney U test, one-way ANOVA, and Kruskal–Wallis test were used to analyze groups of unpaired variables. OS data was available for all 202 patients, while PFS information was available for 147 patients. Survival curves according to different markers were computed using the Kaplan–Meier method, and statistical significance was determined using the Log-rank test. Probability value p <0.05 was considered statistically significant.

## Results

### Characteristics of POLEm and MMRd Tumors

After NGS of 202 tumors, 24 (11.9%) cases with mutations in the exonuclease domain of POLE were identified. Of the 24 POLEm tumors, six (25%) harbored missense mutations at the hotspots Pro286Arg (n = 3) and Val411Leu (n = 3), whereas majority of cases had other variants (**Supplementary Data 2**) (5, 23).

**Figure 1:**
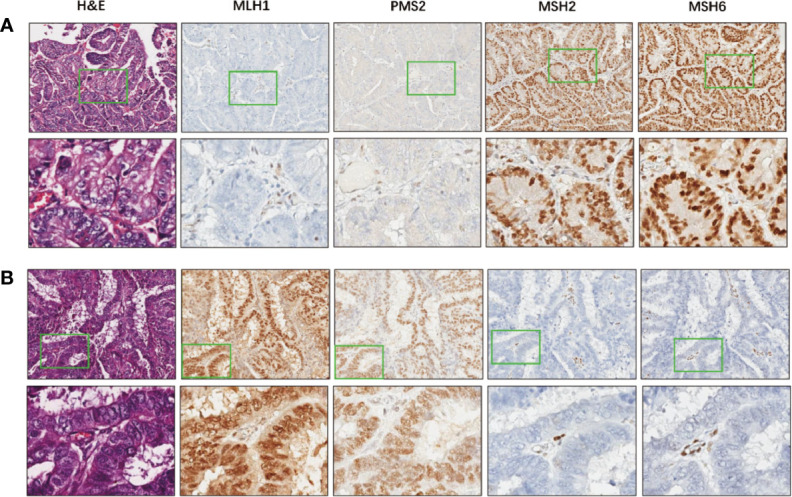
Examples of MMR deficiency in endometrial cancer. **(A)** A representative case that harbors deficiency in MLH1 and PMS2, but proficient in MSH2 and MSH6. Images in the bottom show details of the areas indicated by green boxes in the images above. Magnification: 5× (top) and 200× (bottom). **(B)** A representative case that deficient in MSH2 and MSH6, but proficient in MLH1 and PMS2. Images in the bottom show higher magnification of the areas indicated by green boxes in the images above. Magnification: 5× (top) and 200× (bottom).

IHC for MMR proteins identified 40 (19.8%) patients with MMRd (**Supplementary Data 3**). Thirty one (77.5%) out of the 40 MMRd tumors showed combined protein loss, including 22 (55%) cases lost both MLH1 and PMS2 which form the MutLα complex, and nine (22.5%) cases lost both MSH2 and MSH6 which form the MutSα complex in the MMR system (24). Eight tumors exhibited solitary MSH6 loss, while isolated PMS2 loss was observed in only one tumor. Examples of MMRd cases were shown in **Figure 1**. Out of the 202 tumors, two (1.0%) cases harbored both POLEm and MMRd, which were categorized as POLEm for further analysis, in keeping with a previous study (6).

The clinicopathological features of the 202 ECs were summarized in **Table 1**. The average age at diagnosis in this study population was 56.3 years. POLEm ECs was 4.6 years younger than MMRp patients but the difference was not significant (p = 0.0658). Vast majority of cases (94.1%) were of endometrioid subtype, and there was no difference in histology subtypes across the three groups of ECs (p = 0.865). 197 ECs had information of tumor differentiation, and no significant difference was observed across the three groups. Whereas, compared with MMRp tumors (16.3%), significantly larger proportion of MMRd tumors (34.2%) were poorly differentiated (p = 0.0218). Among the 189 cases who had information on tumor stage, 77.8% were diagnosed at early stages. Overall, there was no difference in stage at diagnosis in the three groups of patients (p = 0.2540). In this study population, majority of patients had no adjuvant therapy. However, significantly larger proportion of women with MMRd (55.3%) had adjuvant treatment compared to MMRp (32.1%) patients (p = 0.0132).

**Table 1:** Clinicopathological characteristics of endometrial cancers regarding POLE and MMR status. One-way ANOVA, Unpaired t test, Chi-square test and Fisher’s Exact test were used and p<0.05 was considered as statistical significance.

	ALL(n = 202)	POLEm (n = 24)	MMRd(n = 38)	MMRp(n = 140)	P value(POLEm, MMRd, MMRp)	P value(POLEm vs MMRp)	P value(MMRd vs MMRp)
**Age at diagnosis,** **Mean (range)**	56.3 (23–82)	51.9 (23–72)	58.3 (41–75)	56.5 (29–82)	0.2278	0.0658	0.9499
**Histology subtypes**	202	24(11.9%)	38(18.8%)	140(69.3%)			
Endometrioid	190	22(11.6%)	36(18.9%)	132(69.5%)	0.865	0.6415	>0.9999
Non-endometrioid	12	2(16.7%)	2(16.7%)	8(66.7%)			
**grade**	197	24(12.2%)	38(19.3%)	135(68.5%)			
1	88	7(7.9%)	16(18.2%)	65(73.9%)	0.2127	0.1187	0.5826
2	69	12(17.4%)	9(13.0%)	48(69.6%)	0.1039	0.2526	0.2407
3	40	5(12.5%)	13(32.5%)	22(55%)	0.0527	0.5628	0.0218
**Stage**	189	21(11.1%)	37(19.6%)	131(69.3%)			
I	143	14(9.8%)	24(16.8%)	105(73.4%)	0.2540(early stages vs late stages)	0.1533(early stages vs late stages)	0.3589(early stages vs late stages)
II	4	0	3(75%)	1(25%)			
III	32	5(15.6%)	9(28.1%)	18(56.2%)			
IV	10	2(20%)	1(10%)	7(70%)			
Total number of early stages	147	14(9.5%)	27(18.4%)	106(72.1%)			
Total number of late stages	42	7(16.7%)	10(23.8%)	25(59.5%)			
**Adjuvant therapy**	202	24(11.9%)	38(18.8%)	140(69.3%)			
Any adjuvant therapy (platinum-based chemotherapy, radiotherapy)	78	12(15.4%)	21(26.9%)	45(57.7%)	0.0163	0.1065	0.0132
No further treatment	124	12(9.7%)	17(13.7%)	95(76.6%)			

POLEm, POLE mutation; MMRd, MMR deficiency; MMRp, MMR proficiency. Data presented as no. (%). Missing data: stage for 13 patients, and grade for five patients.

### Analysis Expression of PD-L1, PD-1, and Density of CD3+ and CD8+ TILs Regarding POLEm, MMRd, and Tumor Differentiation

**Figure 2:**
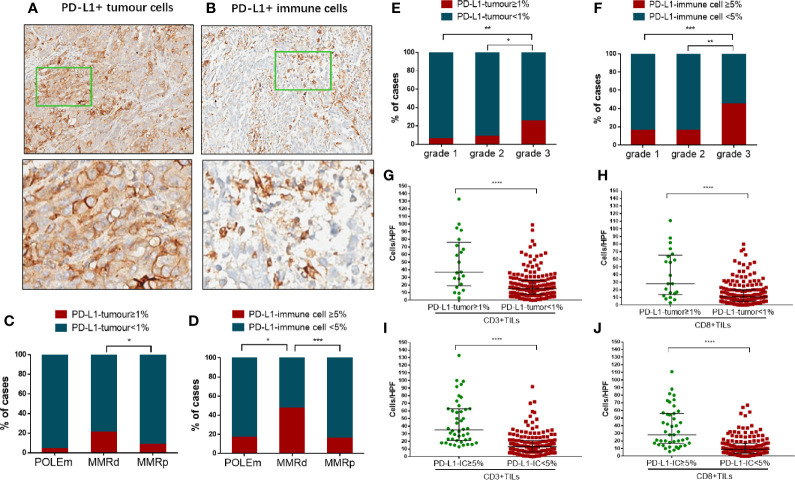
Analysis of PD-L1 expression in tumor cells and immune cells in endometrial cancer. **(A)** A representative case showed PD-L1 positivity in tumor cells. Images in the bottom show details of the areas indicated by green boxes in the images above. Magnification: 5× (top) and 200× (bottom). **(B)** A representative case showed PD-L1 positivity in immune cells. Images in the bottom show details of the areas indicated by green boxes in the images above. Magnification: 5× (top) and 200× (bottom). **(C)** Proportion of cases positive/negative for PD-L1 in tumor cells in POLEm, MMRd and MMRp tumors. **(D)** Proportion of cases positive/negative for PD-L1 in immune cells in POLEm, MMRd and MMRp tumors. **(E)** Proportion of cases positive/negative for PD-L1 in tumor cells in ECs with different differentiation. **(F)** Proportion of cases positive/negative for PD-L1 in immune cells in ECs with different differentiation. **(G)** Comparison of CD3+TILs between cases with positive or negative expression of PD-L1 (tumor cells). **(H)** Comparison of CD8+TILs between cases with positive or negative expression of PD-L1 (tumor cells). **(I)** Comparison of CD3+TILs between cases with positive or negative expression of PD-L1 (immune cells). **(J)** Comparison of CD8+TILs between cases with positive or negative expression of PD-L1 (immune cells). POLEm, POLE mutation; MMRd, MMR deficiency; MMRp, MMR proficiency. Chi-square test and Fisher’s exact test were used, with p <0.05 indicating statistical significance. *P <0.05; **P <0.01; ***P <0.001.

Examples of expression of PD-L1 in TCs and ICs are shown in **Figures 2A, B**. Out of the 202 tumors, only 21 (10.4%) cases showed PD-L1 positivity in TCs, whereas 44 (21.8%) cases exhibited PD-L1 positivity in ICs. Compared with MMRp tumors, significantly larger proportion of MMRd tumors showed positivity in PD-L1 (TC) expression (p = 0.0422, **Figure 2C**). The proportion of PD-L1 (IC) positive cases in the MMRd group was also significantly higher than that in the POLEm group (p = 0.0161) and the MMRp group (p = 0.0001, **Figure 2D**). However, there was no difference in proportion of PD-L1 positive cases between POLEm ECs and MMRp ECs (**Figures 2C, D** **)**. In addition to MMRd status, PD-L1 expression was associated with tumor differentiation in ECs, with high-grade tumors had significantly higher frequency of positive PD-L1 expression in both TCs (grade 3 vs grade 2: p = 0.0264; grade 3 vs grade 1: p = 0.0054) and ICs (grade 3 vs grade 2: p = 0.0015; grade 3 vs grade 1: p = 0.0008) compared to tumors with lower grades (**Figures 2E, F** **)**. Moreover, PD-L1 expression was associated with density of TILs in ECs, with PD-L1 positive tumors exhibited increased number of CD3+ TILs and CD8+TILs (**Figures 2G–J**, p <0.0001).

**Figure 3:**
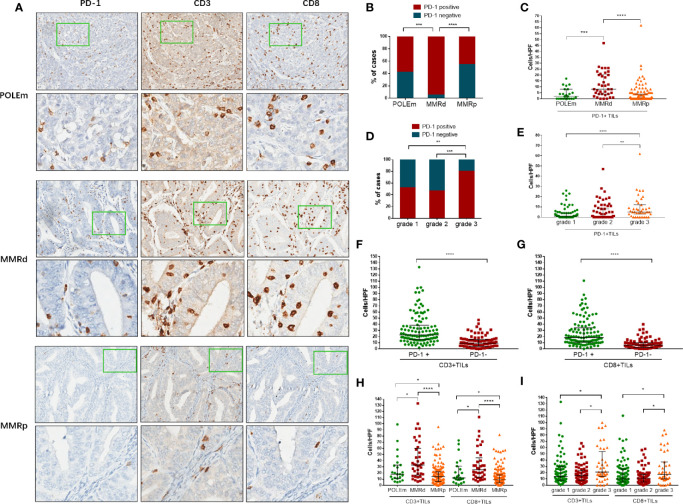
Analysis of PD-1+ TILs, CD3+ TILs and CD8+ TILs in endometrial cancer. **(A)** Representative images of POLEm, MMRd and MMRp tumors. **(B)** Proportion of cases positive/negative for PD-1 in POLEm, MMRd and MMRp tumors. **(C)** Comparison of PD-1+ TILs from POLEm, MMRd and MMRp tumors. **(D)** Proportion of cases positive/negative for PD-1 in grade-1, grade-2 and grade-3 tumors. **(E)** Comparison of PD-1+ TILs from grade-1, grade-2 and grade-3 tumors. **(F)** Comparison of CD3+TILs between PD-1+ and PD-1− tumors. **(G)** Comparison of CD8+TILs between PD-1+ and PD-1− tumors. **(H)** Comparison of CD3+ and CD8+TILs from POLEm, MMRd and MMRp tumors. **(I)** Comparison of CD3+ and CD8+TILs from grade-1, grade-2 and grade-3 tumors. POLEm, POLE mutation; MMRd, MMR deficiency; MMRp, MMR proficiency. TIL, tumor infiltrating lymphocyte. Chi-square test, Fisher’s exact test, Kruskal–Wallis test, and Mann–Whitney test were used, with p <0.05 indicating statistical significance. *P <0.05; **P <0.01; ***P <0.001; ****P <0.0001.

**Figure 3A** shows examples of IHC staining of PD-1, CD3 and CD8 on ECs with POLEm, MMRd or MMRp status. Compared with POLEm tumors (p = 0.0008) and MMRp tumors (p <0.0001), significantly larger proportion of MMRd tumors showed positive PD-1 expression (**Figure 3B**). Tumors with MMRd also exhibited significantly higher number of PD-1+ TILs than POLEm tumors (p = 0.0005) and MMRp tumors (p <0.0001, **Figure 3C**). Whereas, no difference in PD-1 expression was observed between POLEm ECs and MMRp ECs (**Figures 3C, D** **)**.

In addition, as high as 80% of grade 3 tumors were PD-1 positive, the proportion of which was significantly higher than grade 2 tumors (p = 0.0006) and grade 1 tumors (p = 0.0033, **Figure 3D**). We also compared the number of PD-1+TILs in the three groups with different grade, and significantly higher number of PD-1+ TILs were observed in high-grade tumors (grade 3 vs grade 2: p = 0.0018; grade 3 vs grade 1: p <0.0001, **Figure 3E**). We then evaluated the number of CD3+TILs and CD8+TILs in these ECs. Compared to PD-1 negative tumors, significantly higher density of CD3+TILs and CD8+TILs were observed in PD-1 positive tumors (**Figures 3F**, **G**; p <0.0001). MMRd tumors exhibited significantly higher number of CD3+TILs and CD8+TILs than POLEm tumors (CD3: p = 0.0348; CD8: p = 0.0242, **Figure 3H**) and MMRp tumors (CD3: p <0.0001; CD8: p <0.0001, **Figure 3H**). Compared to MMRp ECs, POLEm ECs also showed significantly increased number of CD3+TILs (p = 0.0433) and CD8+TILs (p = 0.0347, **Figure 3H**). In addition to POLE and MMR status, poor differentiation was also associated with increased number of CD3+ and CD8+TILs, with grade 3 tumors exhibiting significantly higher number of CD3+TILs and CD8+TILs than grade 2 tumors (CD3: p = 0.0148; CD8: p = 0.0111, **Figure 3I**) and grade 1 tumors (CD3: p = 0.0286; CD8: p = 0.0463, **Figure 3I**).

Notably, we also observed 22 MMRp cases exhibited high density of CD3+ and/or CD8+TILs (equal to or above the median of that in MMRd tumors), and only six (27.3%) of these MMRp ECs were high-grade tumors (**Figure 3**). Out of the 16 low-grade ECs, eight (50%) were PD-L1 (IC) positive, four (25%) were PD-L1 (TC) positive, and 14 (87.5%) showed positive in PD-1, suggesting a proportion of MMR normal, POLE wild type, and low-grade ECs are potential candidates for PD-1/PD-L1 blockade.

### Prognostic Significance of POLEm, MMRd, PD-1, PD-L1, CD3, and CD8

**Figure 4:**
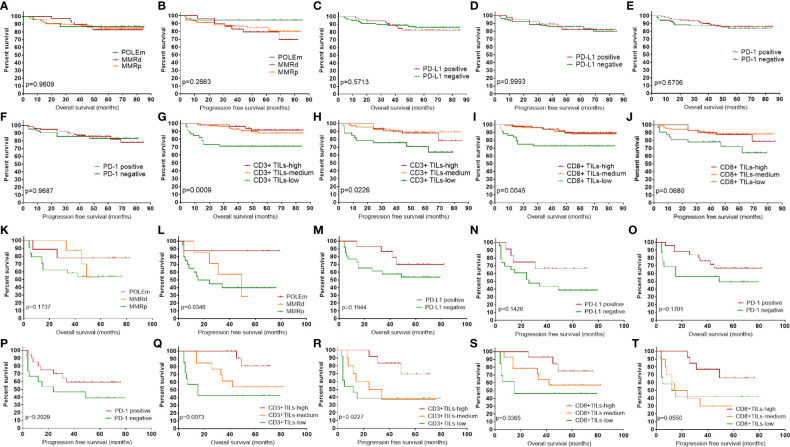
Prognostic impact of POLEm, MMRd, PD-1, PD-L1, CD3 and CD8. **(A)** OS by POLE and MMR status. **(B)** PFS by POLE and MMR status. **(C)** OS by PD-L1 expression. **(D)** PFS by PD-L1 expression. **(E)** OS by PD-1 expression. **(F)** PFS by PD-1 expression. **(G)** OS by density of CD3+TILs. **(H)** PFS by density of CD3+TILs. **(I)** OS by density of CD8+TILs. **(J)** PFS by density of CD8+TILs. **(K)** OS by POLE and MMR status in late-stage ECs. **(L)** PFS by POLE and MMR status in late-stage ECs. **(M)** OS by PD-L1 expression in late-stage ECs. **(N)** PFS by PD-L1 expression in late-stage ECs. **(O)** OS by PD-1 expression in late-stage ECs. **(P)** PFS by PD-1 expression in late-stage ECs. **(Q)** OS by density of CD3+TILs in late-stage ECs. **(R)** PFS by density of CD3+TILs in late-stage ECs. **(S)** OS by density of CD8+TILs in late-stage ECs. **(T)** PFS by density of CD8+TILs in late-stage ECs. POLEm, POLE mutation; MMRd, MMR deficiency; MMRp, MMR proficiency; TIL, tumor infiltrating lymphocyte; OS, overall survival; PFS, progression free survival. Log-rank test was applied, with p <0.05 indicating statistical significance.

We analyzed the prognostic impact of POLEm, MMRd, PD-L1, PD-1, CD3, and CD8 in these ECs. The median follow-up time was 53 months. Patients with POLEm in general had good PFS, with only one patient (Type II tumor, stage III) showed disease progress after platinum-based chemotherapy and radiotherapy. However, the difference in PFS across POLEm, MMRd and MMRp groups was not significant (**Figure 4B**), which may associate with small sample size in the POLEm group. Overall, POLEm, MMRd, PD-L1 and PD-1 showed no significant impact on clinical outcome in this study population (**Figures 4A–F**). To analyze the prognostic impact of CD3 and CD8, patients were divided into three groups according to the density of CD3+ and CD8+TILs. Tumors with TILs count less than the lower-quartile were classified as TILs-low; tumors with TILs count equal to or above the upper-quartile were classified as TILs-high; the rest tumors were classified as TILs-medium. As shown in **Figures 4G, H**, CD3+TILs-high and CD3+TILs-medium ECs had significantly better survival than CD3+TILs-low ECs, but there was no prognostic difference between ECs with high density and medium density of CD3+TILs. A similar pattern of survival was also observed when patients were classified according to density of CD8+TILs (**Figures 4I, J** **)**. As majority of cases in this study were stage I which was associated with good prognosis, we conducted further survival analysis in advanced-stage ECs (n = 42, **Figures 4K–T**). As shown in **Figure 4L**, POLEm ECs had the best PFS across the three groups of patients (POLEm, MMRd and MMRp). PD-L1 and PD-1 showed no significant impact on prognosis (**Figures 4M–P**), while high density of TILs was associated with better clinical outcome in these late-stage ECs (**Figures 4Q–T**).

**Figure 5:**
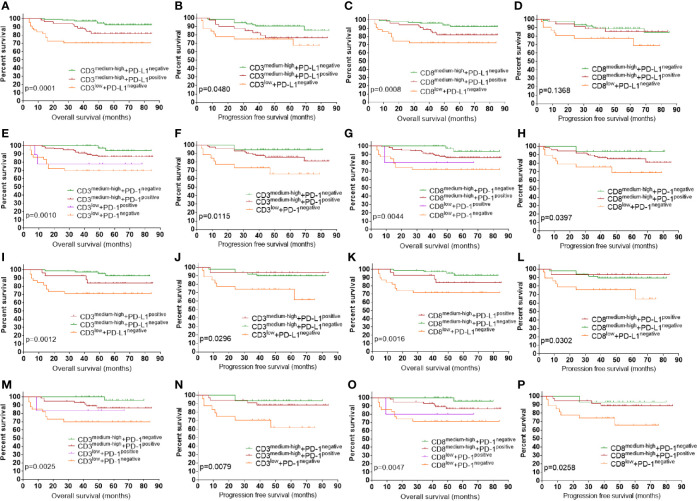
Prognostic impact of combined markers using checkpoint proteins and TILs. **(A)** OS by PD-L1 and CD3+TILs in combination. **(B)** PFS by PD-L1 and CD3+TILs in combination. **(C)** OS by PD-L1 and CD8+TILs in combination. **(D)** PFS by PD-L1 and CD8+TILs in combination. **(E)** OS by PD-1 and CD3+TILs in combination. **(F)** OS by PD-1 and CD3+TILs in combination. **(G)** OS by PD-1 and CD8+TILs in combination. **(H)** PFS by PD-1 and CD8+TILs in combination. **(I)** OS by PD-L1 and CD3+TILs in combination in MMRp ECs. **(J)** PFS by PD-L1 and CD3+TILs in combination in MMRp ECs. **(K)** OS by PD-L1 and CD8+TILs in combination in MMRp EC. **(L)** PFS by PD-L1 and CD8+TILs in combination in MMRp EC. **(M)** OS by PD-1 and CD3+TILs in combination in MMRp EC. **(N)** PFS by PD-1 and CD3+TILs in combination in MMRp EC. **(O)** OS by PD-1 and CD8+TILs in combination in MMRp EC. **(P)** PFS by PD-1 and CD8+TILs in combination in MMRp EC. POLEm, POLE mutation; MMRd, MMR deficiency; MMRp, MMR proficiency. TIL, tumor infiltrating lymphocyte; OS, overall survival. PFS, progression free survival. Log-rank test was applied, with p <0.05 indicating statistical significance.

Since tumors can be stratified to four types of tumor microenvironment (TME) based on T cell infiltration and PD-L1 expression (2, 25), we evaluated the prognostic impact of combined markers using TILs and PD-L1(**Figures 5A–D**). TILs-medium tumors were combined with TILs-high tumors for further survival analysis as they had similar prognosis. Only one case that classified as CD3^low^ + PD-L1^positive^ and CD8^low^ +PD-L1^positive^ was not included in the survival analysis. Classification of patients into four groups according to PD-L1 expression and density of CD3+ TILs showed that CD3^medium-high^ +PD-L1^negative^ group had the best prognosis, followed by the CD3^medium-high^ +PD-L1^positive^ group and the CD3^low^ +PD-L1^negative^ group (**Figures 5A, B** **)**. Stratifying patients to four groups using PD-L1 and CD8+TILs showed a similar pattern of OS (**Figure 5C**), with CD8^medium-high^ +PD-L1^negative^ group had the best prognosis, followed by the CD8^medium-high^+ PD-L1^positive^ group and the CD8^low^ +PD-L1^negative^ group. For PFS, these groups showed a similar pattern, but the difference was not significant (**Figure 5D**). We also assessed the prognostic significance of TILs and PD-1 in combination. Since only four CD3^low^ + PD-L1^positive^ cases and three CD8^low^ +PD-L1^positive^ cases had available PFS information, they were not included in PFS analysis. As shown in **Figures 5E** and **F**, PD-1^negative^ +CD3^medium-high^ group had the best survival. Slightly worse prognosis was observed in the PD-1^positive^ +CD3^medium-high^ group, followed by patients with low CD3 density. Combination of PD-1 and CD8+TILs revealed a similar pattern of prognosis (**Figures 5G, H** **)**. Since POLEm and MMRd tumors are associated with increased density of TILs, we assessed whether MMRp tumors which exhibited wild type POLE and normal MMR expression had similar survival pattern under the same classification. As shown in **Figures 5I–P**, stratification of MMRp ECs using TILs and checkpoint proteins revealed a similar pattern of prognosis as the whole study population. We also evaluated the prognostic impact of combined markers using TILs and checkpoint proteins in advanced-stage ECs (n = 42, **Supplementary Figure 1**). Overall, ECs exhibiting high level of TILs had significantly better prognosis than ECs with lower density of TILs. In addition, low density of TILs and negative expression of PD-L1 in combination was associated with the worst OS (**Supplementary Figures 1A** and **C**), which is similar to the pattern of prognosis as the whole study cohort.

## Discussion

In recent years, immunotherapy which target the immune system rather than cancer cells has emerged as an effective treatment strategy for a variety of cancers. Identification of predictive biomarkers for immunotherapy will help to select patients most likely to benefit from this therapeutic approach. As there are limited treatment options for advanced or relapsed ECs, immunotherapy has potential to improve clinical outcome in carefully chosen patients. ECs with MMRd or POLEm have been suggested as promising candidates for anti PD-1/PD-L1 therapy, whereas recent study also reported that a subgroup of MMRp ECs may also benefit from this therapeutic approach, suggesting additional factors need to be considered for patient selection (7, 26). Being the largest study using full sections to investigate POLEm, MMRd, PD-1, PD-L1, and TILs in ECs, we first characterized the clinicopathological features of POLEm and MMRd ECs in a cohort of 202 cases. Then we evaluated the association of POLEm, MMRd and tumor differentiation with expression of PD-1, PD-L1 and number of TILs. We also assessed the prognostic significance of POLEm, MMRd, PD-1, PD-L1, and TILs in these ECs.

In this patient population, we identified 24 (11.9%) POLEm cases and 40 (19.8%) MMRd cases, which is consistent with previous reports that 7–12% ECs harbor mutations in POLE, and approximately 25% ECs are affected by defective MMR (5, 27–29). In addition, 77.5% of MMRd cases showed combined protein loss, with MLH1 and PMS2 being the most frequently lost MMR proteins, in keeping with previous report by Stelloo et al. (30). It has been reported that POLE mutation was associated with younger age (31). In this patient population, POLEm ECs were diagnosed 4.6 years younger than other patients but the difference was not significant. There was no difference in histology subtype, tumor stage and tumor differentiation across the three groups of ECs, but compared with MMRp ECs, MMRd ECs were significantly associated with higher tumor grade, in line with a previous study by McMeekin et al. (32).

IHC for PD-L1 in these tumors revealed that unlike non-small cell lung cancer and melanoma (33), PD-L1 expression was infrequent in TCs but more common in ICs, in keeping with previous reports by Jones et al. and Howitt et al. (7, 29). There has been limited number of research on expression of PD-1 and PD-L1 in ECs regarding POLE and MMR status (7, 22). Recent studies reported that PD-1 and PD-L1 were overexpressed in both POLE and MSI ECs [MSI and MMRd are highly concordant in ECs (34)], which counterbalanced the increased number of TILs in these tumors (6, 7, 22). In this study, we observed elevated level of CD3+ and CD8+ TILs in both POLEm and MMRd ECs. Whereas, increased number of PD-1+TILs and higher frequency of PD-L1 positivity were associated with MMRd only, not POLEm, suggesting immune suppression *via* upregulation of PD-1 and PD-L1 in only a fraction of these POLEm tumors, other immune checkpoints may play a compensatory role. Overall, these findings support the mechanism that tumors with higher neoantigen loads are more immunogenic and harbor increased TILs, which is counterbalanced by overexpression of various immune checkpoints such as PD-1 and PD-L1 (35).

Indeed, objective response to immunotherapy was demonstrated in POLEm and MMRd ECs (8, 11, 36), and the FDA has recently approved the use of PD-1 blockade for tumors with MMRd/MSI-H, regardless of cancer type (10, 37). Recent studies have shown that POLEm ECs are associated with fewer recurrence, and these patients may not need adjuvant therapy (6, 31, 38). Therefore, it is important to identify MMRp ECs that can benefit from immunotherapy. In the current study, we observed the highest level of CD3+ and CD8+TILs in poorly differentiated ECs. These tumors also had the higher frequency of PD-1 and PD-L1 (IC) positivity compared to low-grade tumors, suggesting high-grade ECs may be promising candidates for immunotherapies targeting the PD-1 pathway, which is consistent with a recent report (29). In addition to grade 3 ECs, a small proportion of low-grade MMRp ECs also exhibited increased density of CD3+ and CD8+ TILs, and were positive for PD-1 and PD-L1 (IC). These results suggest that besides neoantigen loads and tumor differentiation, there are other factors linked with immune responses, and a subgroup of POLE wild type and MMR normal low-grade ECs are potential candidates for immunotherapy.

Survival analysis for these ECs revealed that though MMRd was associated with poor differentiation, a poor prognostic indicator, the clinical outcome of MMRd patients was similar to MMRp patients, consistent with a recent study (32). POLEm showed no impact on OS in this study population but for late-stage ECs, patients with POLEm had significantly better PFS compared to patients with wild type POLE, which is in keeping with previous reports that POLEm was linked to good recurrence free survival (6, 31). In addition, PD-L1 and PD-1 showed no prognostic impact in these EC. Whereas, increased T-cell infiltration was correlated with improved survival, in line with previous reports (25, 39). Although, as single marker, PD-L1 and PD-1 failed to show prognostic significance, classification of ECs to four TME groups using combined markers of PD-L1+TILs or PD-1+TILs showed significant difference in OS across groups. PD-L1^negative^ +TIL^medium-high^ and PD-1^negative^ +TIL^medium-high^ were associated with the best prognosis, which may be explained by the fact that a number of T cells infiltrated to tumors without activation of the PD-1/PD-L1 checkpoint which suppresses T-cell activity. Whereas, other mechanisms of immune evasion may play a dominate role in these tumors (25, 40). Thus, PD-L1^negative^ +TIL^medium-high^ and PD-1^negative^ +TIL^medium-high^ tumors are unlikely to benefit from anti-PD-1/PD-L1 treatment. PD-L1^positive^ +TIL^medium-high^ ECs and PD-1^positive^ +TIL^medium-high^ ECs may be the best candidate to this therapeutic approach as these tumors harboring substantial number of TILs that are switched off through PD-1/PD-L1 pathway. TIL^low^ ECs generally had unfavorable clinical outcome, and are unlikely to response to PD-1/PD-L1 blockade as the lack of T-cell infiltration. However, combination of PD-1/PD-L1 blockade with other approaches that recruit and activate TILs, such as radiotherapy and anti-CTLA-4, would be considered in for these patients (2, 25, 40).

In conclusion, our data has shown that assessment of POLEm and MMRd may be insufficient to identify potential candidates for anti-PD-1/PD-L1 treatment. For better patient selection, additional factors such as tumor differentiation, level of CD3+ and CD8+ TILs, and expression of PD-1 and PD-L1 may need to be taken into consideration. In addition, since the PD-1 pathway is only one of many tumor immune escape mechanisms, understanding the tumor immunity of individual patients may enable design personalized immunotherapy which may combine with other treatment strategies.

## Data Availability Statement

The datasets presented in this study can be found in online repositories. The names of the repository/repositories and accession number(s) can be found in the article/**Supplementary Material**.

## Ethics Statement

The studies involving human participants were reviewed and approved by ethics committee of Sichuan provincial people’s hospital. The patients/participants provided their written informed consent to participate in this study.

## Author Contributions

XX designed and directed the study. XX, DD, HL, DL, HB, YY, BT, KL, and JL performed experiments reported in the study. XX and GX analyzed and interpreted data. XX wrote the manuscript with comments from all authors. All authors contributed to the article and approved the submitted version.

## Funding

This work was supported by Sichuan Provincial People’s Hospital (grant number: 2021ZX02), Sichuan Science and Technology Program (grant number: 2018JY0157), and the National Natural Science Foundation of China (grant number: 81903655).

## Conflict of Interest

The authors declare that the research was conducted in the absence of any commercial or financial relationships that could be construed as a potential conflict of interest.

## Supplementary Material

Supplementary Figure 1 Prognostic impact of combined markers using checkpoint proteins and TILs in advanced-stage ECs. (A) OS by PD-L1 and CD3+TILs in combination. (B) PFS by PD-L1 and CD3+TILs in combination. (C) OS by PD-L1 and CD8+TILs in combination. (D) PFS by PD-L1 and CD8+TILs in combination. (E) OS by PD-1 and CD3+TILs in combination. (F) OS by PD-1 and CD3+TILs in combination. (G) OS by PD-1 and CD8+TILs in combination. (H) PFS by PD-1 and CD8+TILs in combination.

## References

[1] Bray F Ferlay J Soerjomataram I Siegel RL Torre LA Jemal A. Global cancer statistics 2018: GLOBOCAN estimates of incidence and mortality worldwide for 36 cancers in 185 countries. CA Cancer J Clin (2018) 68:394–424. 10.3322/caac.21492 30207593

[2] Kim J Kim S Lee HS Yang W Cho H Chay DB. Prognostic implication of programmed cell death 1 protein and its ligand expressions in endometrial cancer. Gynecol Oncol (2018) 149:381–7. 10.1016/j.ygyno.2018.02.013 29572029

[3] Lortet-Tieulent J Ferlay J Bray F Jemal A. International Patterns and Trends in Endometrial Cancer Incidence, 1978-2013. J Natl Cancer Institute (2018) 110:354–61. 10.1093/jnci/djx214 29045681

[4] Morice P Leary A Creutzberg C Abu-Rustum N Darai E. Endometrial cancer. Lancet (2016) 387:1094–108. 10.1016/S0140-6736(15)00130-0 26354523

[5] Cancer Genome Atlas Research N Kandoth C Schultz N Cherniack AD Akbani R Liu Y. Integrated genomic characterization of endometrial carcinoma. Nature (2013) 497:67–73. 10.1038/nature12113 23636398 PMC3704730

[6] van Gool IC Eggink FA Freeman-Mills L Stelloo E Marchi E de Bruyn M. POLE Proofreading Mutations Elicit an Antitumor Immune Response in Endometrial Cancer. Clin Cancer Res an Off J Am Assoc Cancer Res (2015) 21:3347–55. 10.1158/1078-0432.CCR-15-0057 PMC4627582 25878334

[7] Howitt BE Shukla SA Sholl LM Ritterhouse LL Watkins JC Rodig S. Association of Polymerase e-Mutated and Microsatellite-Instable Endometrial Cancers With Neoantigen Load, Number of Tumor-Infiltrating Lymphocytes, and Expression of PD-1 and PD-L1. JAMA Oncol (2015) 1:1319–23. 10.1001/jamaoncol.2015.2151 26181000

[8] Mehnert JM Panda A Zhong H Hirshfield K Damare S Lane K. Immune activation and response to pembrolizumab in POLE-mutant endometrial cancer. J Clin Invest (2016) 126:2334–40. 10.1172/JCI84940 PMC4887167 27159395

[9] Le DT Uram JN Wang H Bartlett BR Kemberling H Eyring AD. PD-1 Blockade in Tumors with Mismatch-Repair Deficiency. N Engl J Med (2015) 372:2509–20. 10.1056/NEJMoa1500596 PMC4481136 26028255

[10] Ribas A Wolchok JD. Cancer immunotherapy using checkpoint blockade. Science (2018) 359:1350–5. 10.1126/science.aar4060 PMC7391259 29567705

[11] Le DT Durham JN Smith KN Wang H Bartlett BR Aulakh LK. Mismatch repair deficiency predicts response of solid tumors to PD-1 blockade. Science (2017) 357:409–13. 10.1126/science.aan6733 PMC5576142 28596308

[12] Konstantinopoulos PA Luo W Liu JF Gulhan DC Krasner C Ishizuka JJ. Phase II Study of Avelumab in Patients With Mismatch Repair Deficient and Mismatch Repair Proficient Recurrent/Persistent Endometrial Cancer. J Clin Oncol Off J Am Soc Clin Oncol (2019) 37:2786–94. 10.1200/JCO.19.01021 PMC9798913 31461377

[13] Konecny GE. Inhibition of PD-1 and VEGF in microsatellite-stable endometrial cancer. Lancet Oncol (2019) 20:612–4. 10.1016/S1470-2045(19)30079-8 30922730

[14] Ng PC Henikoff S. SIFT: Predicting amino acid changes that affect protein function. Nucleic Acids Res (2003) 31:3812–4. 10.1093/nar/gkg509 PMC168916 12824425

[15] Adzhubei IA Schmidt S Peshkin L Ramensky VE Gerasimova A Bork P. A method and server for predicting damaging missense mutations. Nat Methods (2010) 7:248–9. 10.1038/nmeth0410-248 PMC2855889 20354512

[16] Schwarz JM Rodelsperger C Schuelke M Seelow D. MutationTaster evaluates disease-causing potential of sequence alterations. Nat Methods (2010) 7:575–6. 10.1038/nmeth0810-575 20676075

[17] Baert A Machackova E Coene I Cremin C Turner K Portigal-Todd C. Thorough in silico and in vitro cDNA analysis of 21 putative BRCA1 and BRCA2 splice variants and a complex tandem duplication in BRCA2 allowing the identification of activated cryptic splice donor sites in BRCA2 exon 11. Hum mutation (2018) 39:515–26. 10.1002/humu.23390 29280214

[18] Alipour S Zoghi S Khalili N Hirbod-Mobarakeh A Emens LA Rezaei N. Specific immunotherapy in ovarian cancer: a systematic review. Immunotherapy (2016) 8:1193–204. 10.2217/imt-2016-0034 27605068

[19] Ott PA Bang YJ Berton-Rigaud D Elez E Pishvaian MJ Rugo HS. Safety and Antitumor Activity of Pembrolizumab in Advanced Programmed Death Ligand 1-Positive Endometrial Cancer: Results From the KEYNOTE-028 Study. J Clin Oncol Off J Am Soc Clin Oncol (2017) 35:2535–41. 10.1200/JCO.2017.72.5952 28489510

[20] Ionescu DN Downes MR Christofides A Tsao MS. Harmonization of PD-L1 testing in oncology: a Canadian pathology perspective. Curr Oncol (2018) 25:e209–e16. 10.3747/co.25.4031 PMC6023555 29962847

[21] Eckstein M Cimadamore A Hartmann A Lopez-Beltran A Cheng L Scarpelli M. PD-L1 assessment in urothelial carcinoma: a practical approach. Ann Trans Med (2019) 7:690. 10.21037/atm.2019.10.24 PMC6944605 31930091

[22] Talhouk A Derocher H Schmidt P Leung S Milne K Gilks CB. Molecular Subtype Not Immune Response Drives Outcomes in Endometrial Carcinoma. Clin Cancer Res an Off J Am Assoc Cancer Res (2019) 25:2537–48. 10.1158/1078-0432.CCR-18-3241 30523022

[23] Hussein YR Weigelt B Levine DA Schoolmeester JK Dao LN Balzer BL. Clinicopathological analysis of endometrial carcinomas harboring somatic POLE exonuclease domain mutations. Mod Pathol (2015) 28:505–14. 10.1038/modpathol.2014.143 25394778

[24] Jiricny J. The multifaceted mismatch-repair system. Nat Rev Mol Cell Biol (2006) 7:335–46. 10.1038/nrm1907 16612326

[25] Teng MW Ngiow SF Ribas A Smyth MJ. Classifying Cancers Based on T-cell Infiltration and PD-L1. Cancer Res (2015) 75:2139–45. 10.1158/0008-5472.CAN-15-0255 PMC4452411 25977340

[26] Crumley S Kurnit K Hudgens C Fellman B Tetzlaff MT Broaddus R. Identification of a subset of microsatellite-stable endometrial carcinoma with high PD-L1 and CD8+ lymphocytes. Mod Pathol (2019) 32:396–404. 10.1038/s41379-018-0148-x 30291344 PMC6395512

[27] Black D Soslow RA Levine DA Tornos C Chen SC Hummer AJ. Clinicopathologic significance of defective DNA mismatch repair in endometrial carcinoma. J Clin Oncol Off J Am Soc Clin Oncol (2006) 24:1745–53. 10.1200/JCO.2005.04.1574 16549821

[28] Church DN Briggs SE Palles C Domingo E Kearsey SJ Grimes JM. DNA polymerase epsilon and delta exonuclease domain mutations in endometrial cancer. Hum Mol Genet (2013) 22:2820–8. 10.1093/hmg/ddt131 PMC3690967 23528559

[29] Jones NL Xiu J Rocconi RP Herzog TJ Winer IS. Immune checkpoint expression, microsatellite instability, and mutational burden: Identifying immune biomarker phenotypes in uterine cancer. Gynecol Oncol (2019) 156:393–9. 10.1016/j.ygyno.2018.04.095 31882243

[30] Stelloo E Jansen AML Osse EM Nout RA Creutzberg CL Ruano D. Practical guidance for mismatch repair-deficiency testing in endometrial cancer. Ann Oncol Off J Eur Soc Med Oncol (2017) 28:96–102. 10.1093/annonc/mdw542 27742654

[31] Church DN Stelloo E Nout RA Valtcheva N Depreeuw J ter Haar N. Prognostic significance of POLE proofreading mutations in endometrial cancer. J Natl Cancer Institute (2015) 107:402. 10.1093/jnci/dju402 PMC4301706 25505230

[32] McMeekin DS Tritchler DL Cohn DE Mutch DG Lankes HA Geller MA. Clinicopathologic Significance of Mismatch Repair Defects in Endometrial Cancer: An NRG Oncology/Gynecologic Oncology Group Study. J Clin Oncol Off J Am Soc Clin Oncol (2016) 34:3062–8. 10.1200/JCO.2016.67.8722 PMC5012715 27325856

[33] Taube JM Klein A Brahmer JR Xu H Pan X Kim JH. Association of PD-1, PD-1 ligands, and other features of the tumor immune microenvironment with response to anti-PD-1 therapy. Clin Cancer Res an Off J Am Assoc Cancer Res (2014) 20:5064–74. 10.1158/1078-0432.CCR-13-3271 PMC4185001 24714771

[34] Powell MA. Immunohistochemistry to determine mismatch repair-deficiency in endometrial cancer: the appropriate standard. Ann Oncol Off J Eur Soc Med Oncol (2017) 28:9–10. 10.1093/annonc/mdw628 28177429

[35] Llosa NJ Cruise M Tam A Wicks EC Hechenbleikner EM Taube JM. The vigorous immune microenvironment of microsatellite instable colon cancer is balanced by multiple counter-inhibitory checkpoints. Cancer Discovery (2015) 5:43–51. 10.1158/2159-8290.CD-14-0863 25358689 PMC4293246

[36] Santin AD Bellone S Buza N Choi J Schwartz PE Schlessinger J. Regression of Chemotherapy-Resistant Polymerase epsilon (POLE) Ultra-Mutated and MSH6 Hyper-Mutated Endometrial Tumors with Nivolumab. Clin Cancer Res an Off J Am Assoc Cancer Res (2016) 22:5682–7. 10.1158/1078-0432.CCR-16-1031 PMC5135588 27486176

[37] Nebot-Bral L Brandao D Verlingue L Rouleau E Caron O Despras E. Hypermutated tumours in the era of immunotherapy: The paradigm of personalised medicine. Eur J Cancer (2017) 84:290–303. 10.1016/j.ejca.2017.07.026 28846956

[38] Van Gool IC Rayner E Osse EM Nout RA Creutzberg CL Tomlinson IPM. Adjuvant Treatment for POLE Proofreading Domain-Mutant Cancers: Sensitivity to Radiotherapy, Chemotherapy, and Nucleoside Analogues. Clin Cancer Res an Off J Am Assoc Cancer Res (2018) 24:3197–203. 10.1158/1078-0432.CCR-18-0266 29559562

[39] de Jong RA Leffers N Boezen HM ten Hoor KA van der Zee AG Hollema H. Presence of tumor-infiltrating lymphocytes is an independent prognostic factor in type I and II endometrial cancer. Gynecol Oncol (2009) 114:105–10. 10.1016/j.ygyno.2009.03.022 19411095

[40] Kim JM Chen DS. Immune escape to PD-L1/PD-1 blockade: seven steps to success (or failure). Ann Oncol Off J Eur Soc Med Oncol (2016) 27:1492–504. 10.1093/annonc/mdw217 27207108

