# Supplementary figures and images for: POLE and Mismatch Repair Status, Checkpoint Proteins and Tumor-Infiltrating Lymphocytes in Combination, and Tumor Differentiation: Identify Endometrial Cancers for Immunotherapy
Source: Front Oncol. 2021 Mar 19;11:640018. doi: 10.3389/fonc.2021.640018 (PMC8017289; doi:10.3389/fonc.2021.640018)

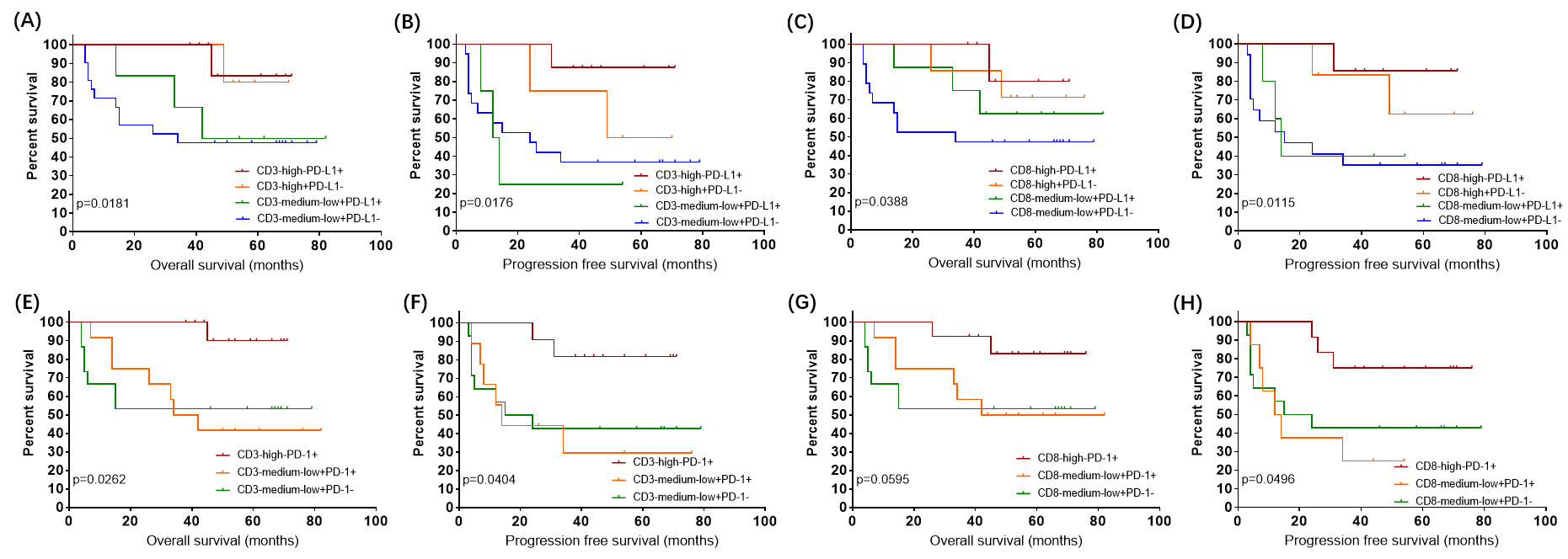

Supplement: Supplementary Figure 1 — Prognostic impact of combined markers using checkpoint proteins and TILs in advanced-stage ECs. (A) OS by PD-L1 and CD3+TILs in combination. (B) PFS by PD-L1 and CD3+TILs in combination. (C) OS by PD-L1 and CD8+TILs in combination. (D) PFS by PD-L1 and CD8+TILs in combination. (E) OS by PD-1 and CD3+TILs in combination. (F) OS by PD-1 and CD3+TILs in combination. (G) OS by PD-1 and CD8+TILs in combination. (H) PFS by PD-1 and CD8+TILs in combination. [file Image_1.tif]
