# Supplementary material for: POLE and Mismatch Repair Status, Checkpoint Proteins and Tumor-Infiltrating Lymphocytes in Combination, and Tumor Differentiation: Identify Endometrial Cancers for Immunotherapy
Source: Front Oncol. 2021 Mar 19;11:640018. doi: 10.3389/fonc.2021.640018 (PMC8017289; doi:10.3389/fonc.2021.640018)
Supplement: Supplementary file 2 [file Table_1.docx]

| Antibodies | Clone | Manufacture | Dilution |
| --- | --- | --- | --- |
| MLH1 | ES05 | ZSGB-BIO | prediluted |
| MSH2 | RED2 | ZSGB-BIO | prediluted |
| MSH6 | EP49 | ZSGB-BIO | prediluted |
| PMS2 | M0R4G | ZSGB-BIO | prediluted |
| CD3 | SP7 | MXB Biotechnologies | prediluted |
| CD8 | SP16 | MXB Biotechnologies | prediluted |
| PD-1 | UMAB199 | ZSGB-BIO | prediluted |
| PD-L1 | SP142 | ZSGB-BIO | prediluted |

**Supplementary data 1. List of antibodies used in IHC**
