# Supplementary material for: POLE and Mismatch Repair Status, Checkpoint Proteins and Tumor-Infiltrating Lymphocytes in Combination, and Tumor Differentiation: Identify Endometrial Cancers for Immunotherapy
Source: Front Oncol. 2021 Mar 19;11:640018. doi: 10.3389/fonc.2021.640018 (PMC8017289; doi:10.3389/fonc.2021.640018)
Supplement: Supplementary file 3 [file Table_2.docx]

| **Case NO.** | **Gene Region** | **Predicted Protein Variants** |
| --- | --- | --- |
| ET13-15 | exonic | exon13:c.1253C>T:p.P418L, |
| ET13-17 | exonic | exon12:c.1220G>A:p.C407Y |
|  | exonic | exon12:c.1184G>A:p.G395E |
|  | exonic | exon10:c.943G>A:p.E315K |
| ET13-21 | exonic | exon10:c.998G>A:p.C333Y |
|  | exonic | exon13:c.1346C>T:p.T449M, |
|  | exonic | exon12:c.1184G>A:p.G395E, |
| ET13-22 | exonic | exon13:c.1297G>A:p.G433S, |
| ET13-25 | exonic | exon13:c.1280C>T:p.A427V, |
|  | exonic | exon14:c.1439C>T:p.A480V, |
|  | exonic | exon13:c.1336C>T:p.R446W, |
| ET13-30 | exonic | exon14:c.1366G>C:p.A456P, |
| ET13-36 | exonic | exon9:c.857C>G:p.P286R, |
| ET13-4 | exonic | exon10:c.998G>A:p.C333Y, |
| ET13-9 | splicing | exon13:c.1227-1G>C, |
|  | exonic | exon13:c.1235A>G:p.K412R, |
| ET14-18 | exonic | exon13:c.1306C>T:p.P436S, |
| ET14-19 | exonic | exon13:c.1336C>T:p.R446W, |
| ET14-22 | exonic | exon13:c.1327G>A:p.D443N, |
| ET14-24 | exonic | exon13:c.1264C>T:p.H422Y, |
| ET14-41 | splicing | exon10:c.910-6G>A, |
| ET14-44 | exonic | exon14:c.1396A>G:p.T466A, |
| ET14-45 | exonic | exon14:c.1435T>C:p.F479L, |
|  | exonic | exon14:c.1439C>T:p.A480V, |
|  | exonic | exon13:c.1336C>T:p.R446W, |
|  | exonic | exon14:c.1403A>C:p.Y468S, |
| ET14-51 | exonic | exon9:c.857C>G:p.P286R, |
| ET14-9 | exonic | exon13:c.1231G>T:p.V411L |
| ET15-1 | exonic | exon13:c.1231G>A:p.V411M, |
| ET15-35 | exonic | exon13:c.1231G>T:p.V411L |
| ET15-46 | exonic | exon9:c.857C>G:p.P286R, |
| ET15-47 | exonic | exon9:c.845C>T:p.P282L, |
|  | exonic | exon14:c.1394C>T:p.A465V, |
|  | exonic | exon14:c.1457C>T:p.P486L, |
|  | exonic | exon10:c.966C>G:p.F322L, |
| ET15-76 | exonic | exon10:c.991C>T:p.P331S, |
| ET16-18 | exonic | exon13:c.1231G>T:p.V411L |

**Supplementary data 2.** **Cases harbouring likely pathogenic mutations of POLE**
