# Supplementary material for: POLE and Mismatch Repair Status, Checkpoint Proteins and Tumor-Infiltrating Lymphocytes in Combination, and Tumor Differentiation: Identify Endometrial Cancers for Immunotherapy
Source: Front Oncol. 2021 Mar 19;11:640018. doi: 10.3389/fonc.2021.640018 (PMC8017289; doi:10.3389/fonc.2021.640018)
Supplement: Supplementary file 4 [file Table_3.docx]

| **Case NO.** | **Detected MMR protein loss by IHC** | | | |
| --- | --- | --- | --- | --- |
|  | **MLH1** | **PMS2** | **MSH2** | **MSH6** |
| ET16-15 | MLH1- | PMS2- |  |  |
| ET16-19 | MLH1- | PMS2- |  |  |
| ET16-24 |  |  | MSH2- | MSH6- |
| ET16-35 |  |  |  | MSH6- |
| ET16-41 |  |  | MSH2- | MSH6- |
| ET16-43 | MLH1- | PMS2- |  |  |
| ET16-51 |  |  | MSH2- | MSH6- |
| ET16-57 |  |  |  | MSH6- |
| ET16-63 | MLH1- | PMS2- |  |  |
| ET15-13 | MLH1- | PMS2- |  |  |
| ET15-14 | MLH1- | PMS2- |  |  |
| ET15-15 | MLH1- | PMS2- |  |  |
| ET15-17 | MLH1- | PMS2- |  |  |
| ET15-23 | MLH1- | PMS2- |  |  |
| ET15-24 |  |  | MSH2- | MSH6- |
| ET15-30 |  |  |  | MSH6- |
| ET15-34 | MLH1- | PMS2- |  |  |
| ET15-47 | MLH1- | PMS2- |  |  |
| ET15-50 | MLH1- | PMS2- |  |  |
| ET15-55 |  |  |  | MSH6- |
| ET15-57 |  |  |  | MSH6- |
| ET15-65 |  |  |  | MSH6- |
| ET15-72 |  |  | MSH2- | MSH6- |
| ET14-1 | MLH1- | PMS2- |  |  |
| ET14-8 | MLH1- | PMS2- |  |  |
| ET14-35 |  |  | MSH2- | MSH6- |
| ET14-40 |  |  |  | MSH6- |
| ET14-48 |  | PMS2- |  |  |
| ET14-55 |  |  | MSH2- | MSH6- |
| ET13-2 |  |  |  | MSH6- |
| ET13-6 | MLH1- | PMS2- |  |  |
| ET13-7 |  |  | MSH2- | MSH6- |
| ET13-12 | MLH1- | PMS2- |  |  |
| ET13-16 | MLH1- | PMS2- |  |  |
| ET13-18 | MLH1- | PMS2- |  |  |
| ET13-21 | MLH1- | PMS2- |  |  |
| ET13-23 | MLH1- | PMS2- |  |  |
| ET13-26 | MLH1- | PMS2- |  |  |
| ET13-27 |  |  | MSH2- | MSH6- |
| ET13-33 | MLH1- | PMS2- |  |  |

**Supplementary data3. Cases harbouring MMR protein deficiency**
